# The Effect of Preoperative Antiplatelet Therapy on Early Postoperative Rehemorrhage and Outcomes in Patients With Spontaneous Intracranial Hematoma

**DOI:** 10.3389/fnagi.2021.681998

**Published:** 2021-07-02

**Authors:** Junhua Yang, Qingyuan Liu, Shaohua Mo, Kaiwen Wang, Maogui Li, Jun Wu, Pengjun Jiang, Shuzhe Yang, Rui Guo, Yi Yang, Jiaming Zhang, Yang Liu, Yong Cao, Shuo Wang

**Affiliations:** ^1^Department of Neurosurgery, Beijing Tiantan Hospital, Capital Medical University, Beijing, China; ^2^China National Clinical Research Center for Neurological Diseases, Beijing, China; ^3^Center of Stroke, Beijing Institute for Brain Disorders, Beijing, China; ^4^Beijing Key Laboratory of Translational Medicine for Cerebrovascular Disease, Beijing, China

**Keywords:** spontaneous intracranial hematoma, stroke, surgery, antiplatelet, complications

## Abstract

**Background and Purpose:**

The effect of antiplatelet therapy (APT) on early postoperative rehemorrhage and outcomes of patients with spontaneous intracerebral hemorrhage (ICH) is still unclear. This study is to evaluate the effect of preoperative APT on early postoperative rehemorrhage and outcomes in ICH patients.

**Methods:**

This was a multicenter cohort study. ICH patients undergoing surgery were divided into APT group and no antiplatelet therapy (nAPT) group according to whether patients received APT or not. Chi-square test, *t*-test, and Mann–Whitney *U* test were used to compare the differences in variables, postoperative rehematoma, and outcomes between groups. Multivariate logistics regression analysis was used to correct for confounding variables, which were different in group comparison.

**Results:**

One hundred fifty ICH patients undergoing surgical treatment were consecutively included in this study. Thirty five (23.33%) people were included in the APT group, while 115 (76.67%) people were included in the nAPT group. The incidence of early postoperative rehemorrhage in the APT group was significantly higher than that in the nAPT group (25.7% VS 10.4%, *p* = 0.047 < 0.05). After adjustment for age, ischemic stroke history, and ventricular hematoma, preoperative APT had no significant effect on early postoperative rehemorrhage (*p* = 0.067). There was no statistical difference between the two groups in early poorer outcomes (*p* = 0.222) at 14 days after surgery. After adjustment for age, ischemic stroke history, and ventricular hematoma, preoperative APT also had no significant effect on early poorer modified Rankin Scale (mRS) (*p* = 0.072).

**Conclusion:**

In conclusion, preoperative APT appears to be safe and have no significant effect on early postoperative rehematoma and outcomes in ICH patients.

## Introduction

The incidence of spontaneous intracranial hemorrhage (ICH) increases with age (An et al., 2017). According to reports (Cordonnier et al., 2018; Luzzi et al., 2019), the mortality rate of ICH can be as high as 50%. Although there is no specific treatment for ICH patients, emergency surgery may reduce the mortality rate of ICH patients (Wu et al., 2020). Surgical methods mainly include craniectomy with hematoma evacuation, endoscopic surgery, minimally invasive puncture, and thrombolysis (Luzzi et al., 2019). These procedures can effectively reduce the intracranial pressure and hematoma volume of ICH patients (Luzzi et al., 2019; Wu et al., 2020). However, surviving ICH patients may be at risk of postoperative complications, especially postoperative rehemorrhage, which may seriously affect the prognosis of patients (Yu et al., 2016).

According to report, antiplatelet therapy (APT) is widely used in neurosurgery patients at the time of consultation (Fiaschi et al., 2020). Although the impact of APT on tumor patients can be reduced by delaying surgery (Rahman et al., 2015), patients with ICH usually do not have enough time to completely eliminate the impact of APT before surgery. Previous studies mainly focused on the effect of APT on hematoma volume or hematoma expansion in ICH patients (Sansing et al., 2009; Khan et al., 2017). The evidence for the effect of preoperative APT on early postoperative rehemorrhage and prognosis of ICH patients is still insufficient.

Thus, to evaluate the impact of APT on early postoperative rehemorrhage and outcomes in ICH patients, we designed this cohort study.

## Materials and Methods

This was a multicenter cohort study that has been registered on the website of the Chinese Clinical Trial Registry (trial registration number: ChiCTR1900024406)^1^ and has received the support of the Institutional Review Board of Beijing Tiantan Hospital, Capital Medical University (reference number: KY2019-096-02).

### Study Population

The population of the current study was consecutively enrolled from a retrospective and a prospective ICH cohort according the inclusion and exclusion criteria. Patients in the retrospective cohort were those who received surgical treatment in Beijing Tiantan Hospital, Capital Medical University between January 1, 2015 and July 31,2019, while patients in prospective cohort were those who received surgical treatment in Beijing Tiantan Hospital, Beijing Chaoyang Hospital, Beijing Friendship Hospital, Beijing Anzhen Hospital, Beijing Shunyi District Hospital, Beijing Pinggu District Hospital, or Guangzhou Red Cross Hospital between August 1, 2019 and December 31, 2019. All patients’ informed consent forms were signed by themselves or their family members.

### Inclusion and Exclusion Criteria

Inclusion criteria were as follows: (1) patients older than 18 years old, (2) patients diagnosed as ICH, and (3) patients received surgery treatment within 7 days after the onset of symptoms.

Exclusion criteria were as follows: (1) ICH caused by secondary causes such as trauma, tumor, moyamoya disease, aneurysm, venous thrombosis, cerebral ischemic infarction, etc.; (2) patients accompanied by primary and secondary coagulopathy; (3) patients accompanied by malignant tumor or liver or renal dysfunction; (4) patients receiving any form of anticoagulation therapy within 7 days before surgery; (5) patients with incomplete clinical data; and (6) patients without informed consent.

### Surgery

All ICH patients in this study received standard care in accordance with guidelines (Hemphill et al., 2015) and received craniotomy, endoscopic, or minimally invasive surgery to remove hematoma within 7 days after the onset of symptoms. Two or three experienced neurosurgeons jointly decided on the surgical plan and performed the operation. The selection of surgical methods has been described in our previous reports (Wu et al., 2020), and the surgical indications included supratentorial hematoma greater than 30 ml, subtentorial hematoma greater than 10 ml, midline displacement larger than 1 cm, and brain herniation. In addition, APT before the onset of ICH was not considered an absolute contraindication to surgery. However, the patients with insufficient platelet count or decreased platelet activity may be treated with platelet transfusion. After operation, the first follow-up computerized tomography (CT) scan was routinely performed in 24 h. Then, follow-up CTs were performed every 2–3 days or when patients had new neurological symptoms.

### Data Collection

The collected characteristics of ICH patients included (1) demographic characteristics, such as patients’ age and gender; (2) vascular risk factors, such as patients’ history of smoking and alcohol; (3) medical history, such as patients’ history of hypertension, diabetes, coronary heart disease, ischemic stroke, cerebral hemorrhage, and antiplatelet agent types (including aspirin, clopidogrel, or aspirin plus clopidogrel); (4) radiography characteristics analyzed by three neurosurgeons who were blinded to the information of enrolled patients, such as hematoma side, localization (the basal ganglia, thalamus, internal capsule, brain stem, and cerebellum were defined as deep; the frontal, temporal, parietal, and occipital were defined as lobar), hematoma volume calculated by *A* × *B* × *C*/2 method (Kothari et al., 1996), hemorrhage expansion, ventricular hematoma, and subarachnoid hemorrhage; (5) surgery information, such as time from symptom onset to surgery, surgical approach, intraoperative blood loss, and hematoma evacuation rate; (6) laboratory characteristics, such as platelets (PLT) count, international normalized ratio (INR), activated partial thromboplastin time (APTT), and platelet transfusion; and (7) functional status, such as modified Rankin Scale (mRS) and mortality.

### Outcomes and Definition

Taking into account the effective time of antiplatelet drugs (Cahill et al., 2005; Hornor et al., 2018), outcomes were determined according to the mRS and all-cause mortality at 14 days after surgery.

The definition of postoperative hemorrhage is as follows: compared with the previous postoperative CT scan, the volume of the hematoma increased by >33% (the ICH volume decreased significantly after minimally invasive surgery), or the CT scan at the follow-up after the operation found that the volume of hematoma was completely high-density shadows that appeared again in the excised primary site (Wu et al., 2017; Shen et al., 2018).

Preoperative APT is defined as the continuous administration of aspirin (100 mg), clopidogrel (75 mg), or aspirin plus clopidogrel for more than 7 days due to coronary heart disease, cerebral infarction, or other ischemic lesions before the operation, and the interruption time is less than 7 days (Ford, 2015; Hornor et al., 2018).

### Statistical Analysis

Platelets count and mRS values were transformed into categorical variables. Categorical variables were described by percentage, and continuous variables were described by means (standard deviations) or medians (quartiles) when appropriate. Categorical variables were analyzed by chi-square test. According to whether continuous variables conformed to normal distribution, the *t* or Mann–Whitney *U* test were used for analysis. Multivariate logistics regression analysis was used to correct confounding factors whose *p*-value was less than 0.05 in the comparison between groups. All statistical tests were performed by SPSS statistical software (IBM, version 26). A *p*-value of <0.05 on both sides was considered statistically significant.

## Results

### Study Population

From January 1, 2015 to July 31, 2019, 1,548 patients were diagnosed with ICH in Beijing Tiantan Hospital, Capital Medical University. Among them, 176 adult patients received surgery treatment; 38 patients were excluded because they were diagnosed with aneurysm, arteriovenous malformation, moyamoya disease, etc.; eight patients were excluded due to receiving anticoagulation therapy within 7 days before surgery; and eight patients were excluded due to incomplete data. In the end, 122 patients in the retrospective cohort were included in this study. And according to the inclusion and exclusion criteria, from January 2019 to December 2019, a total of 28 patients in the prospective cohort were consecutively enrolled into current study. Totally, 150 ICH patients were involved in this study.

### Baseline Characteristics

The enrolled population was divided into APT group and no antiplatelet therapy (nAPT) group according to whether they accept APT or not. Thirty five (23.33%) people were included in the APT group, while 115 (76.67%) people were included in the nAPT group. The baseline characteristics of the two groups are shown in Table 1. As shown, the age of patients in the APT group was older than that in the nAPT group (*p* = 0.001); patients in the APT group were more likely to have a history of ischemic stroke than patients in the nAPT group (*p* = 0.000); patients in the APT group were also more prone to ventricular hemorrhage when ICH occurred than patients in the APT group (*p* = 0.000) (Table 1). In addition, there was no statistical difference between the two groups in variables of hematoma volume, hemorrhage expansion, surgical methods, intraoperative blood loss, hematoma evacuation rate, postoperative residual hematoma volume, APTT, and other factors (Table 1).

**TABLE 1:** Patient’s baseline characteristics according to preoperative APT.

Variables	nAPT (*N* = 115, 76.67%)	APT (*N* = 35, 23.33%)	*P*-value
**Demographic characteristics**			
Gender			0.175
Male	97 (84.35%)	26 (74.29%)	
Female	18 (15.65%)	9 (25.71%)	
Age (years)	48.39 ± 12.713	57.29 ± 13.024	0.001
**Vascular risk factors**			
Smoking	59 (51.30%)	12 (34.29%)	0.077
Alcohol	73 (63.48%)	16 (45.71%)	0.061
**Medical history**			
Hypertension	107 (93.04%)	32 (91.43%)	0.719
Diabetes	11 (9.57%)	6 (17.14%)	0.230
Coronary heart disease	6 (5.22%)	5 (14.29%)	0.129
Ischemic stroke history	5 (4.35%)	14 (40.00%)	0.000
Cerebral hemorrhage history	4 (3.49%)	3 (8.57%)	0.355
**Imaging**			
Side			0.709
Left	60 (52.17%)	17 (48.57%)	
Right	55 (47.83%)	18 (51.43%)	
Localization			0.096
Lobar	21 (18.26%)	11 (31.43%)	
Deep	94 (81.74%)	24 (68.57%)	
Hematoma volume (ml)	46.37 (31.36, 58.58)	54.11 (33.36, 76.90)	0.077
Hemorrhage expansion	43 (37.39%)	10 (28.57%)	0.629
Ventricular hematoma	52 (45.22%)	29 (82.86%)	0.000
Subarachnoid hemorrhage	25 (21.74%)	7 (20.00%)	0.826
**Surgery**			
Time from symptom onset to surgery (h)	45 (22, 93)	32 (15, 60)	0.149
Surgery			0.368
Craniotomy	50 (43.48%)	19 (54.29%)	
Endoscopic surgery	10 (8.70%)	4 (11.43%)	
Minimally invasive surgery	55 (47.83%)	12 (34.29%)	
Intraoperative blood loss (ml)	200 (100, 400)	300 (200, 500)	0.053
Rate of hematoma evacuation (%)	92.30 (75.90, 96.77)	89.00 (70.42, 93.40)	0.226
Postoperative residual hematoma volume (ml)	2.63 (1.03, 12.79)	4.33 (1.12, 21.14)	0.645
**Laboratory test**			
PLT 10^9/l			0.102
<125	2 (1.74%)	3 (8.57%)	
125–350	107 (93.04%)	29 (82.8%)	
>350	6 (5.22%)	3 (8.57%)	
INR	0.96 (0.89, 1.02)	0.99 (0.94, 1.04)	0.087
APTT (s)	26.00 (23.50, 28.48)	26.80 (24.20, 30.40)	0.109
Platelet transfusion	7 (6.09%)	5 (14.29%)	0.152
Neurological condition at admission			1.000
mRS (0–3)	12 (10.43%)	4 (11.43%)	
mRS (4–5)	103 (89.57%)	31 (88.57%)	

*APT, antiplatelet therapy; nAPT, no antiplatelet therapy; *N*, number; PLT, platelets; INR, international normalized ratio; mRS, postoperative modified Rankin Scale; APTT, activated partial thromboplastin time.*

### Differences in Postoperative Rehemorrhage and Outcomes Between Groups

Nine (25.7%) patients in the APT group developed early postoperative rehematoma, and 12 (10.4%) patients in the nAPT group developed early postoperative rehematoma (Table 2). As Table 2 shows, the incidence of early postoperative rehemorrhage in the APT group was significantly higher than that in the nAPT group (*p* = 0.047 < 0.05). However, there was no significant difference (0.286) in postoperative hematoma expansion volume between the two groups.

**TABLE 2:** The association between preoperative APT and postoperative rehemorrhage or outcomes.

	nAPT (*N* = 115, 76.67%)	APT (*N* = 35, 23.33%)	*P*-value
Postoperative rehemorrhage	12 (10.43%)	9 (25.71%)	0.047
Postoperative hematoma expansion volume (ml)	15.76 (3.26, 24.48)	8.50 (3.80, 13.99)	0.286
Outcomes			0.222
mRS (0–3)	19 (16.52%)	9 (25.71%)	
mRS (4–5)	94 (81.74%)	23 (65.72%)	
Mortality	2 (1.74%)	3 (8.57%)	

*APT, antiplatelet therapy; nAPT, no antiplatelet therapy; *N*, number; mRS, postoperative modified Rankin Scale.*

**FIGURE 1:**
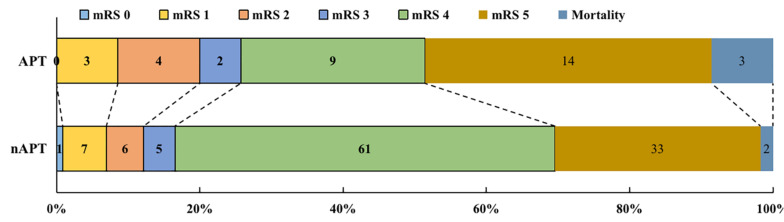
The outcomes of patients with ICH after surgery. APT, antiplatelet therapy; nAPT, no antiplatelet therapy; mRS, modified Rankin Scale.

In the APT group, 9 (25.7%) patients had a good early prognosis, while 26 (74.3%) patients had a poor early prognosis, of which 3 (8.6%) patients died early after surgery (Figure 1). In the nAPT group, 19 patients (16.5%) had a better early prognosis, while 96 patients (83.5%) had a poor early prognosis and two patients (1.7%) died earlier after surgery (Figure 1). As Table 2 shows, there was no statistical difference between the two groups in early poorer outcomes (*p* = 0.222) at 14 days after surgery.

### Effect of APT on Postoperative Rehemorrhage and Outcomes

After adjusting for age, history of ischemic stroke, and ventricular hematoma variables, preoperative APT had no significant effect on early postoperative rehemorrhage [*p* = 0.067; 95% confidence interval (CI), 3.046 (0.926, 10.031)], as shown in Table 3.

**TABLE 3:** The effects of preoperative APT on postoperative rehemorrhage after adjustment.

Variables	Odds ratio (95% CI)	*P*-value
APT	3.046 (0.926, 10.031)	0.067
Age (years)	0.998 (0.962, 1.036)	0.920
Ischemic stroke history	0.941 (0.237, 3.735)	0.931
Ventricular hematoma	1.034 (0.368, 2.906)	0.949

*APT, antiplatelet therapy; CI, confidence interval.*

As shown in Table 4, preoperative APT also had no significant effect on early poorer outcomes [*p* = 0.072; 95% CI, 0.324 (0.095, 1.105)] after adjusting for age, history of ischemic stroke, and ventricular hematoma.

**TABLE 4:** The effects of APT on outcomes after adjustment.

Variables	Odds ratio (95% CI)	*P-*value
APT	0.324 (0.095, 1.105)	0.072
Age (years)	0.991 (0.958, 1.026)	0.620
Ischemic stroke history	1.160 (0.279, 4.813)	0.838
Ventricular hematoma	3.792 (1.408, 10.213)	0.008

*APT, antiplatelet therapy; CI, confidence interval.*

## Discussion

Postoperative rehemorrhage seriously affects the prognosis of ICH patients. Previous research reported that postoperative rehemorrhage is an independent risk factor of poor outcome (Ren et al., 2018). However, there is still a lack of evidence in neurosurgery to support the hypothesis that preoperative APT may increase the risk of early postoperative rehemorrhage and affect early prognosis. So based on the hypothesis, we designed this study.

The results of the current study found that although the proportion of early postoperative rehemorrhage in ICH patients receiving APT is higher than that of patients not receiving APT, after adjusting for age, history of ischemic stroke, and ventricular hematoma, preoperative APT did not significantly increase the probability of early postoperative rehemorrhage in ICH patients. Similarly, preoperative APT did not significantly affect early poorer prognosis (mRS and mortality) of ICH patients undergoing surgery.

The results of current study were consistent with previous studies on the effects of APT on neurosurgery. Hanalioglu et al. (2020) studied 1,346 cases of intracranial tumor surgery and reported that discontinued APT before surgery and continued APT during perioperative period all did not significantly increase the probability of postoperative rehematoma. The same conclusion was also obtained in patients with traumatic brain injury undergoing craniotomy or craniectomy (Greuter et al., 2019). Although there were reports that preoperative APT was an independent risk factor for postoperative rehematoma in patients with ICH, the population of this study excluded deep ICH, which was the majority part of ICH (Biffi et al., 2010). This may cause bias in the results of their study. Besides, in another observational study on lobar and deep ICH, APT did not affect the recurrence of ICH after ICH (Weimar et al., 2011). Although its population included ICH patients who have not undergone surgery, it showed that APT had no significant effect on rehematoma, which indirectly proved the conclusion of the current study.

However, some studies in the field of non-neurosurgery have reached the opposite conclusion. In 2016, a retrospective research analyzed more than 4,500 patients undergoing thyroid surgery and found that patients who received preoperative APT had a significantly higher risk of postoperative hematoma than patients who did not undergo preoperative APT (*p* < 0.01) (Oltmann et al., 2016). Noteworthy, the population in this study took aspirin at a dose of 325 mg, which is higher than the low dose (100 mg) for cerebrovascular disease. Therefore, the conclusion of this study may not be applicable to patients with cerebrovascular disease. Subsequently, Luni et al. (2017) reported in a meta-analysis of cardiac surgery patients that patients who did not discontinue aspirin in time (>5 days) suffered a higher risk of postoperative rehemorrhage (*p* = 0.04). It seems that preoperative aspirin abuse increases the risk of postoperative rehemorrhage. However, its *p*-value was at a critical point, and the studies included in this meta-analysis have a moderate degree of heterogeneity (Luni et al., 2017), which may lead to controversial results.

Although previous studies have shown that preoperative APT does not significantly affect the occurrence of postoperative rehemorrhage, platelet transfusion is still used before surgery in ICH patients to prevent hemorrhage and postoperative rehemorrhage by improving platelet function. However, majority of evidences (Naidech et al., 2012; Suzuki et al., 2014; Baschin et al., 2017) supporting the efficacy of platelet transfusion on ICH come from observational studies, and such studies (Naidech et al., 2012; Suzuki et al., 2014; Baschin et al., 2017) are susceptible to various confounding factors and cannot accurately reflect the impact of platelet transfusion on ICH. In 2016, a randomized controlled trial conducted by PATCH showed that platelet transfusion after ICH in people receiving APT increased the patient’s chance of death (Baharoglu et al., 2016). Subsequently, the research of Al-Shahi Salman et al. (2018) also reached the same conclusion, and some researchers (Magid-Bernstein et al., 2021) proposed that the poor efficacy after platelet transfusion may be related to the mismatch of ABO blood type. In the current study, only a small percentage of ICH patients received platelet transfusion before surgery and no significant difference was observed between the two groups. Therefore, this study cannot clarify the effect of platelet transfusion on postoperative rehemorrhage in ICH patients. More researches need to be implemented to clarify the effect of platelet transfusion in ICH patients.

On the other hand, some previous observational studies have shown that preoperative APT was a risk factor for poorer prognosis and mortality (Roquer et al., 2005; Yang et al., 2014; Won et al., 2017; Maas et al., 2018; Würtz et al., 2018), but this result was not observed in our study. The group comparison and correction analysis in our study showed preoperative APT did not affect the early outcome of ICH patients. This may be related to different study populations and observation periods from previous studies. Unlike previous studies (Roquer et al., 2005; Yang et al., 2014; Won et al., 2017; Maas et al., 2018; Würtz et al., 2018), our study only included ICH patients undergoing surgical treatment and the observation period was shorter. Recently, two studies (Khan et al., 2017; Franco et al., 2020) have challenged previous conclusions that preoperative APT is a risk factor for poor prognosis and death in ICH patients. Khan et al. (2017) analyzed the data of 82,576 ICH patients and found that single APT did not significantly increase the risk of in-hospital mortality. Franco et al. (2020) found a similar conclusion that previous APT was not an independent predictor of in-hospital mortality, which was exactly the conclusion of our research.

This study explored the effect of preoperative APT on early postoperative rehemorrhage and prognosis in ICH patients undergoing surgery and may provide some evidence for the clinical application of APT in ICH patients. In addition, this is a multi-center cohort study, which could avoid certain case limitations and bias. The research still has certain shortcomings. The current study is only based on the effective time of antiplatelet drugs to determine the study observation time, but did not detect the patient’s platelet function during this period, which may have a certain impact on the accuracy of the results. Besides, this study was non-random and the included population was small, which inevitably had a certain impact on the accuracy of the results. Therefore, large randomized controlled trials are needed to verify the accuracy of this conclusion.

## Conclusion

Preoperative APT appears to be safe and has no significant effect on early postoperative rehematoma and outcomes in ICH patients.

## Data Availability Statement

The original contributions presented in the study are included in the article/supplementary material, further inquiries can be directed to the corresponding author/s.

## Ethics Statement

The studies involving human participants were reviewed and approved by the Institutional Review Board of Beijing Tiantan Hospital, Capital Medical University. The patients/participants provided their written informed consent to participate in this study.

## Author Contributions

SW made significant contributions in the conceptualization of the study. JY, QL, SM, and ML made significant contributions in the methodology of the study. JY, SM, KW, and SY made significant contributions in the formal analysis and investigation. JY made significant contributions in the original draft preparation. JW, RG, YY, JZ, and YL made significant contributions in reviewing and editing of the manuscript. PJ, YC, and SW made significant contributions in the supervision of the study. All authors contributed to the article and approved the submitted version.

## Conflict of Interest

The authors declare that the research was conducted in the absence of any commercial or financial relationships that could be construed as a potential conflict of interest.

## Footnotes

1 http://www.chictr.org.cn/
